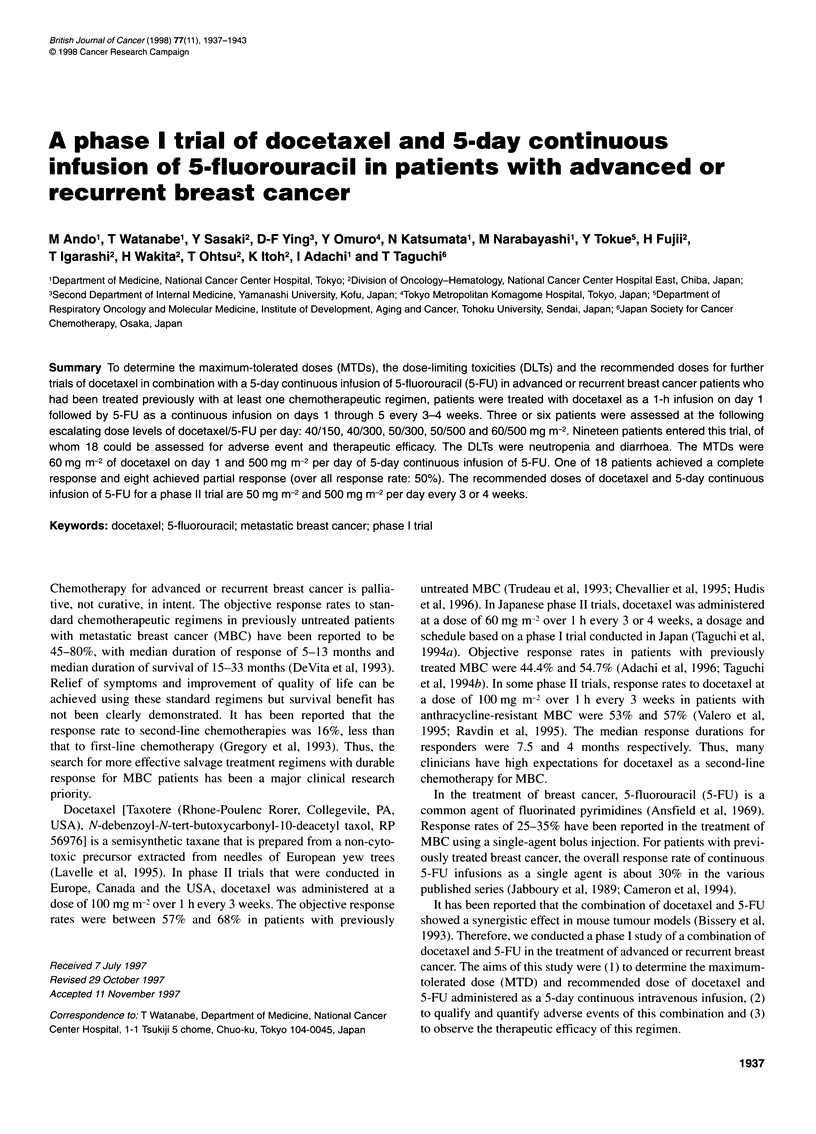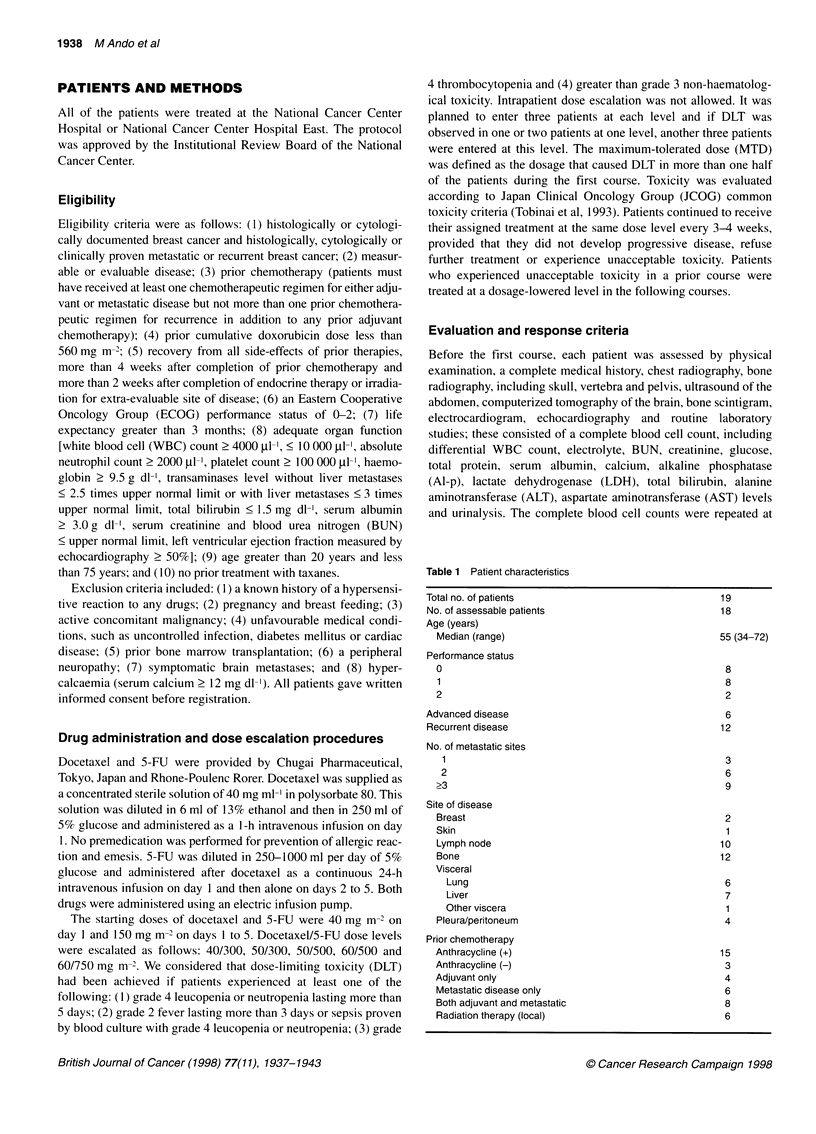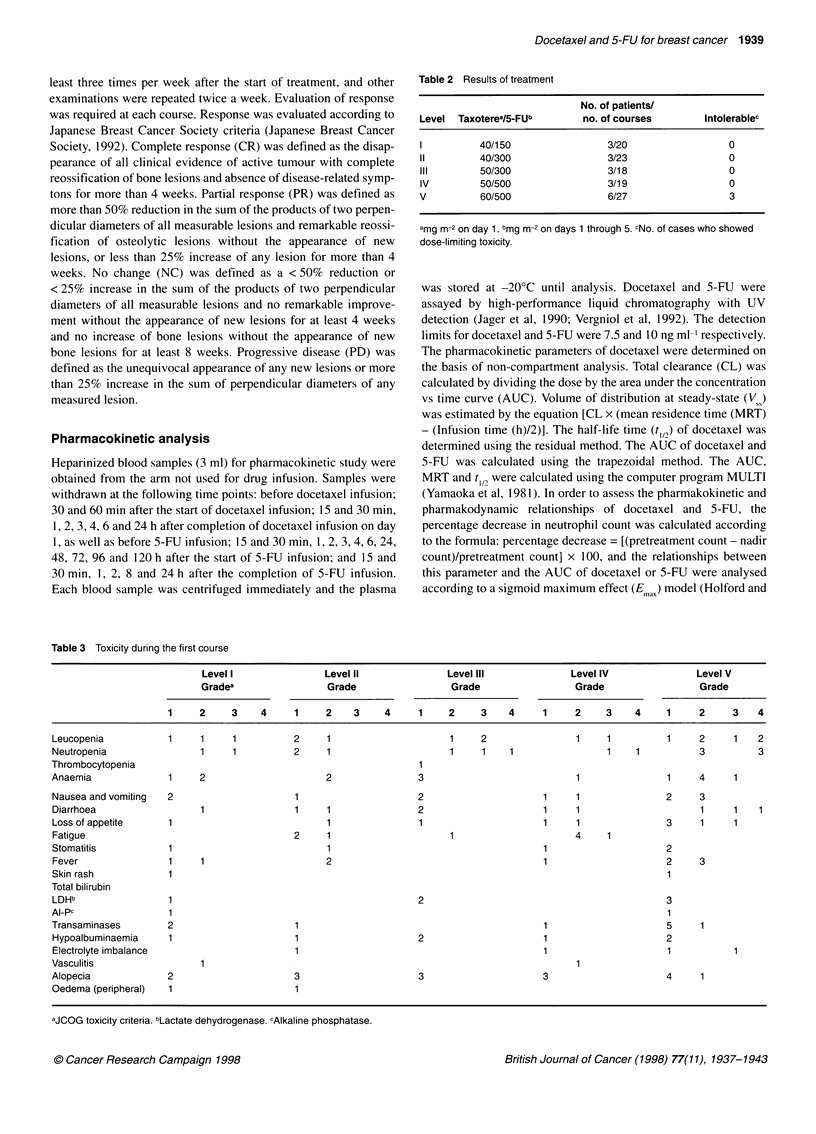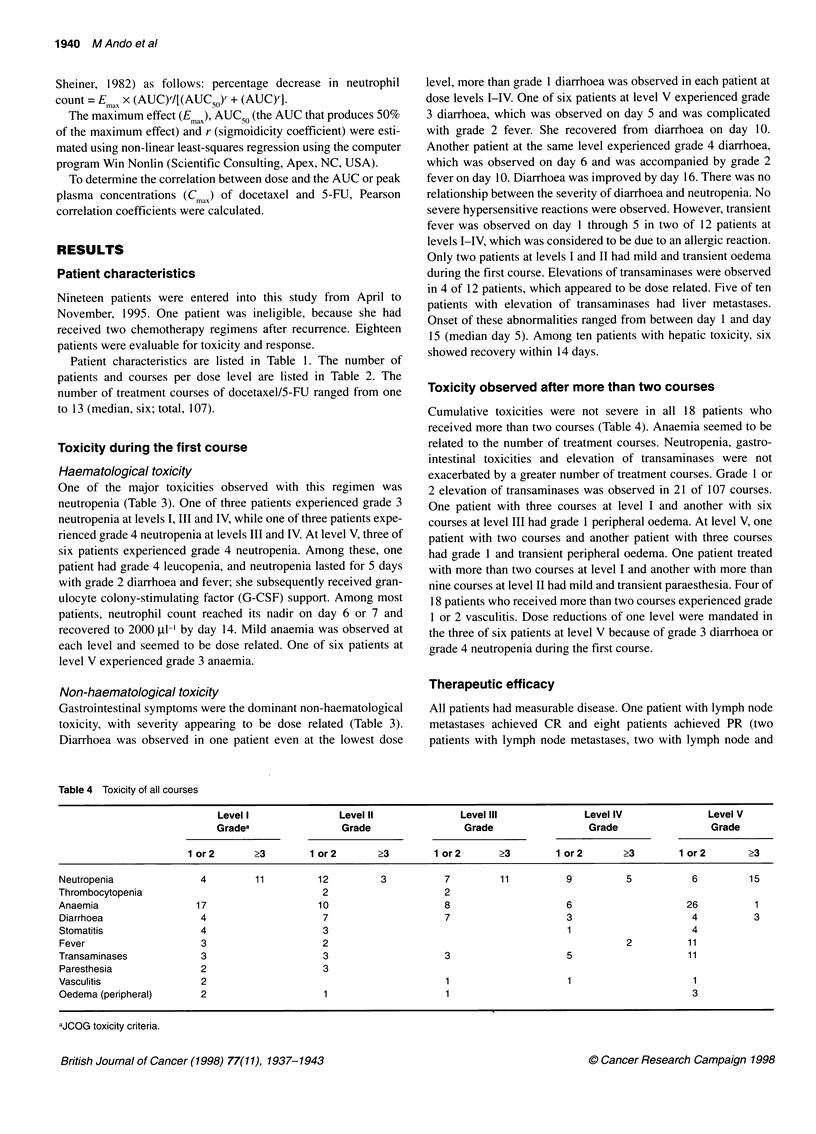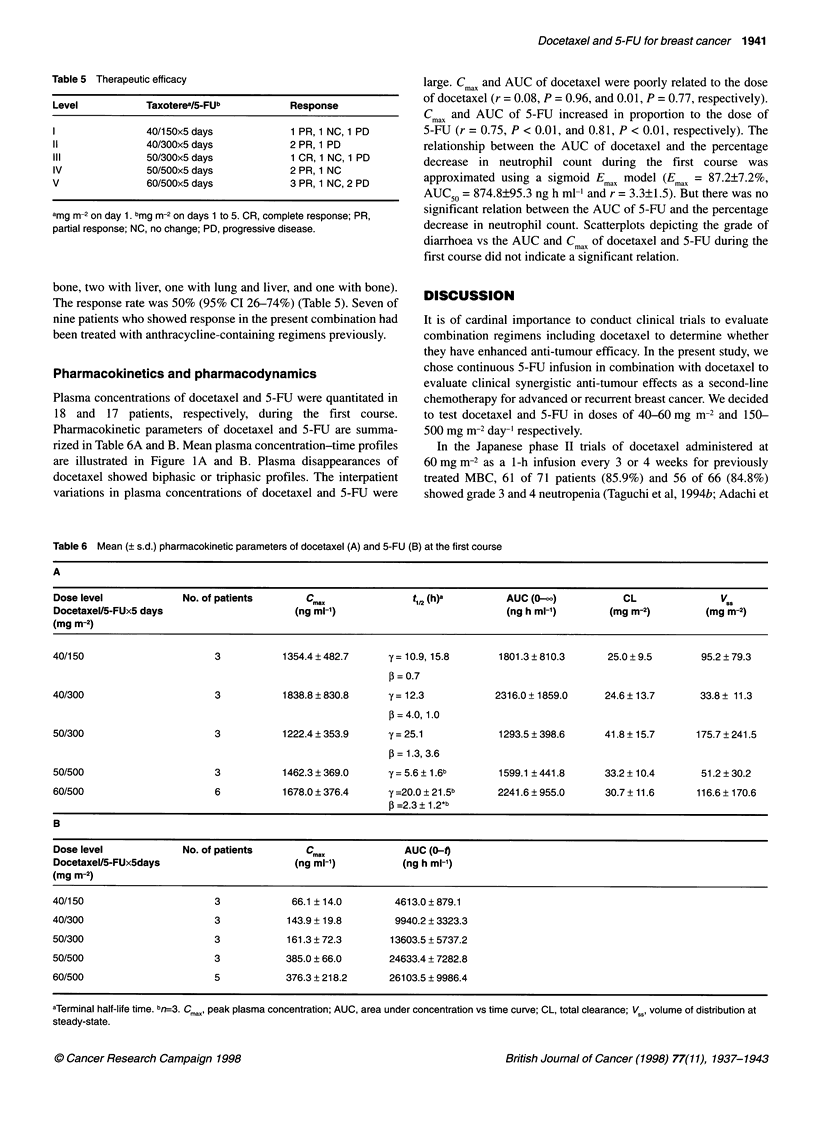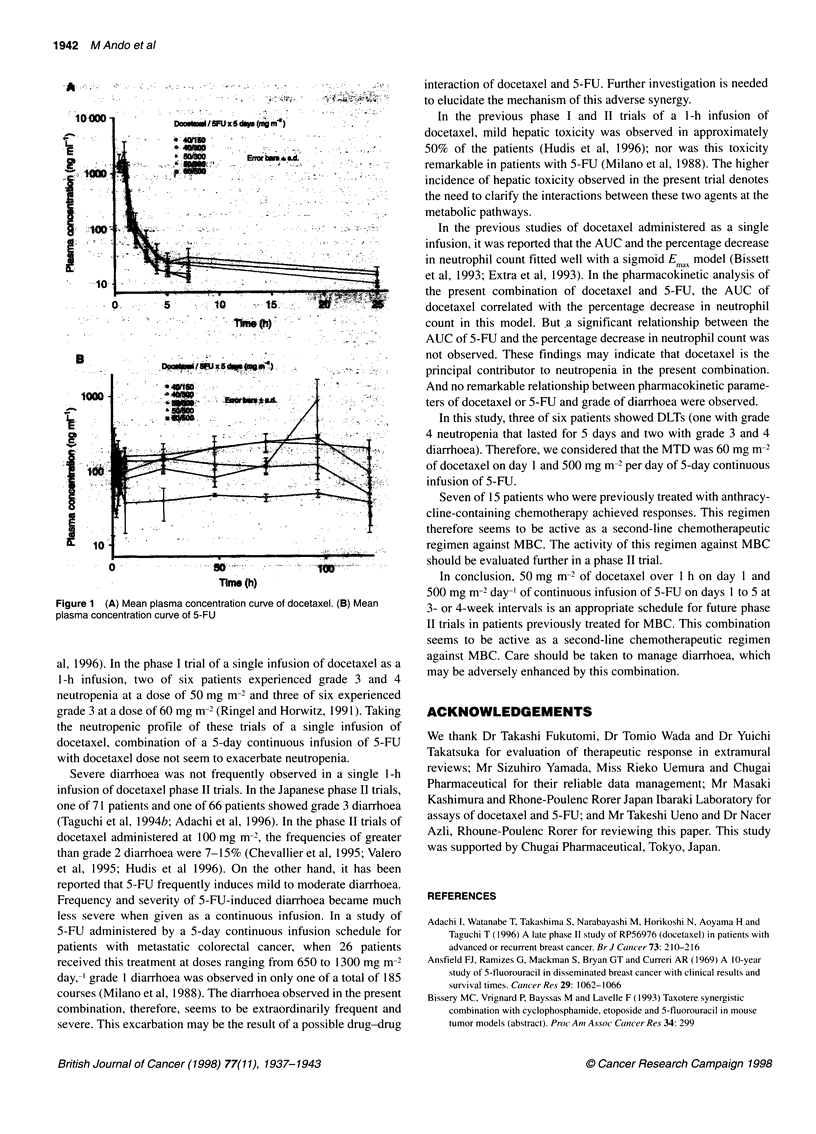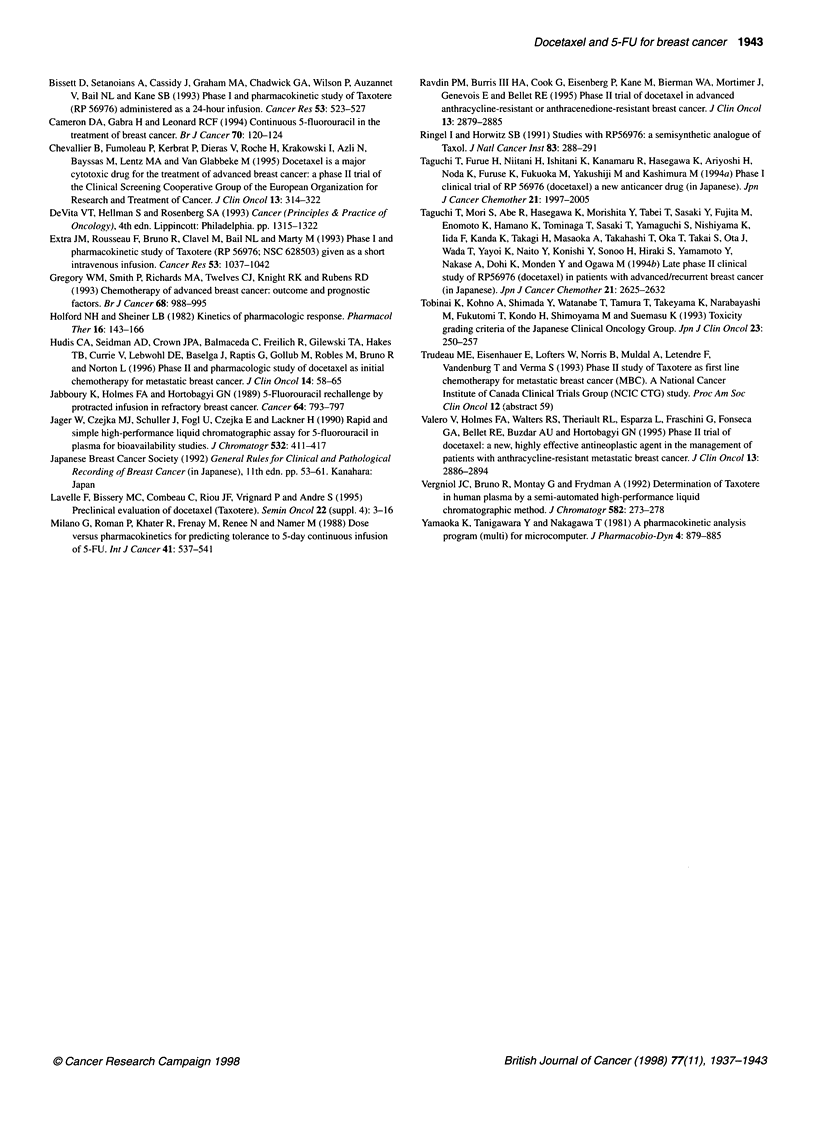# A phase I trial of docetaxel and 5-day continuous infusion of 5-fluorouracil in patients with advanced or recurrent breast cancer.

**DOI:** 10.1038/bjc.1998.321

**Published:** 1998-06

**Authors:** M. Ando, T. Watanabe, Y. Sasaki, D. F. Ying, Y. Omuro, N. Katsumata, M. Narabayashi, Y. Tokue, H. Fujii, T. Igarashi, H. Wakita, T. Ohtsu, K. Itoh, I. Adachi, T. Taguchi

**Affiliations:** Department of Medicine, National Cancer Center Hospital, Tokyo, Japan.

## Abstract

To determine the maximum-tolerated doses (MTDs), the dose-limiting toxicities (DLTs) and the recommended doses for further trials of docetaxel in combination with a 5-day continuous infusion of 5-fluorouracil (5-FU) in advanced or recurrent breast cancer patients who had been treated previously with at least one chemotherapeutic regimen, patients were treated with docetaxel as a 1-h infusion on day 1 followed by 5-FU as a continuous infusion on days 1 through 5 every 3-4 weeks. Three or six patients were assessed at the following escalating dose levels of docetaxel/5-FU per day: 40/150, 40/300, 50/300, 50/500 and 60/500 mg m(-2). Nineteen patients entered this trial, of whom 18 could be assessed for adverse event and therapeutic efficacy. The DLTs were neutropenia and diarrhoea. The MTDs were 60 mg m(-2) of docetaxel on day 1 and 500 mg m(-2) per day of 5-day continuous infusion of 5-FU. One of 18 patients achieved a complete response and eight achieved partial response (over all response rate: 50%). The recommended doses of docetaxel and 5-day continuous infusion of 5-FU for a phase II trial are 50 mg m(-2) and 500 mg m(-2) per day every 3 or 4 weeks.


British Journal of Cancer (1998) 77(11), 1937–1943
© 1998 Cancer Research Campaign

### A phase I trial of docetaxel and 5-day continuous infusion of 5-fluorouracil in patients with advanced or recurrent breast cancer

**M Ando[1], T Watanabe[1], Y Sasaki[2], D-F Ying[3], Y Omuro[4], N Katsumata[1], M Narabayashi[1], Y Tokue[5], H Fujii[2], T Igarashi[2], H Wakita[2], T Ohtsu[2], K Itoh[2], I Adachi[1] and T Taguchi[6]**

[1]Department of Medicine, National Cancer Center Hospital, Tokyo; [2]Division of Oncology–Hematology, National Cancer Center Hospital East, Chiba, Japan; [3]Second Department of Internal Medicine, Yamanashi University, Kofu, Japan; [4]Tokyo Metropolitan Komagome Hospital, Tokyo, Japan; [5]Department of Respiratory Oncology and Molecular Medicine, Institute of Development, Aging and Cancer, Tohoku University, Sendai, Japan; [6]Japan Society for Cancer Chemotherapy, Osaka, Japan

**Summary** To determine the maximum-tolerated doses (MTDs), the dose-limiting toxicities (DLTs) and the recommended doses for further trials of docetaxel in combination with a 5-day continuous infusion of 5-fluorouracil (5-FU) in advanced or recurrent breast cancer patients who had been treated previously with at least one chemotherapeutic regimen, patients were treated with docetaxel as a 1-h infusion on day 1 followed by 5-FU as a continuous infusion on days 1 through 5 every 3–4 weeks. Three or six patients were assessed at the following escalating dose levels of docetaxel/5-FU per day: 40/150, 40/300, 50/300, 50/500 and 60/500 mg m$^{-2}$. Nineteen patients entered this trial, of whom 18 could be assessed for adverse event and therapeutic efficacy. The DLTs were neutropenia and diarrhoea. The MTDs were 60 mg m$^{-2}$ of docetaxel on day 1 and 500 mg m$^{-2}$ per day of 5-day continuous infusion of 5-FU. One of 18 patients achieved a complete response and eight achieved partial response (over all response rate: 50%). The recommended doses of docetaxel and 5-day continuous infusion of 5-FU for a phase II trial are 50 mg m$^{-2}$ and 500 mg m$^{-2}$ per day every 3 or 4 weeks.

**Keywords:** docetaxel; 5-fluorouracil; metastatic breast cancer; phase I trial

Chemotherapy for advanced or recurrent breast cancer is palliative, not curative, in intent. The objective response rates to standard chemotherapeutic regimens in previously untreated patients with metastatic breast cancer (MBC) have been reported to be 45–80%, with median duration of response of 5–13 months and median duration of survival of 15–33 months (DeVita et al, 1993). Relief of symptoms and improvement of quality of life can be achieved using these standard regimens but survival benefit has not been clearly demonstrated. It has been reported that the response rate to second-line chemotherapies was 16%, less than that to first-line chemotherapy (Gregory et al, 1993). Thus, the search for more effective salvage treatment regimens with durable response for MBC patients has been a major clinical research priority.

Docetaxel [Taxotere (Rhone-Poulenc Rorer, Collegevile, PA, USA), *N*-debenzoyl-*N*-tert-butoxycarbonyl-10-deacetyl taxol, RP 56976] is a semisynthetic taxane that is prepared from a non-cytotoxic precursor extracted from needles of European yew trees (Lavelle et al, 1995). In phase II trials that were conducted in Europe, Canada and the USA, docetaxel was administered at a dose of 100 mg m$^{-2}$ over 1 h every 3 weeks. The objective response rates were between 57% and 68% in patients with previously untreated MBC (Trudeau et al, 1993; Chevallier et al, 1995; Hudis et al, 1996). In Japanese phase II trials, docetaxel was administered at a dose of 60 mg m$^{-2}$ over 1 h every 3 or 4 weeks, a dosage and schedule based on a phase I trial conducted in Japan (Taguchi et al, 1994*a*). Objective response rates in patients with previously treated MBC were 44.4% and 54.7% (Adachi et al, 1996; Taguchi et al, 1994*b*). In some phase II trials, response rates to docetaxel at a dose of 100 mg m$^{-2}$ over 1 h every 3 weeks in patients with anthracycline-resistant MBC were 53% and 57% (Valero et al, 1995; Ravdin et al, 1995). The median response durations for responders were 7.5 and 4 months respectively. Thus, many clinicians have high expectations for docetaxel as a second-line chemotherapy for MBC.

In the treatment of breast cancer, 5-fluorouracil (5-FU) is a common agent of fluorinated pyrimidines (Ansfield et al, 1969). Response rates of 25–35% have been reported in the treatment of MBC using a single-agent bolus injection. For patients with previously treated breast cancer, the overall response rate of continuous 5-FU infusions as a single agent is about 30% in the various published series (Jabboury et al, 1989; Cameron et al, 1994).

It has been reported that the combination of docetaxel and 5-FU showed a synergistic effect in mouse tumour models (Bissery et al, 1993). Therefore, we conducted a phase I study of a combination of docetaxel and 5-FU in the treatment of advanced or recurrent breast cancer. The aims of this study were (1) to determine the maximum-tolerated dose (MTD) and recommended dose of docetaxel and 5-FU administered as a 5-day continuous intravenous infusion, (2) to qualify and quantify adverse events of this combination and (3) to observe the therapeutic efficacy of this regimen.

*Received 7 July 1997*
*Revised 29 October 1997*
*Accepted 11 November 1997*

*Correspondence to:* T Watanabe, Department of Medicine, National Cancer Center Hospital, 1-1 Tsukiji 5 chome, Chuo-ku, Tokyo 104-0045, Japan

### PATIENTS AND METHODS

All of the patients were treated at the National Cancer Center Hospital or National Cancer Center Hospital East. The protocol was approved by the Institutional Review Board of the National Cancer Center.

### Eligibility

Eligibility criteria were as follows: (1) histologically or cytologically documented breast cancer and histologically, cytologically or clinically proven metastatic or recurrent breast cancer; (2) measurable or evaluable disease; (3) prior chemotherapy (patients must have received at least one chemotherapeutic regimen for either adjuvant or metastatic disease but not more than one prior chemotherapeutic regimen for recurrence in addition to any prior adjuvant chemotherapy); (4) prior cumulative doxorubicin dose less than 560 mg $m^{-2}$; (5) recovery from all side-effects of prior therapies, more than 4 weeks after completion of prior chemotherapy and more than 2 weeks after completion of endocrine therapy or irradiation for extra-evaluable site of disease; (6) an Eastern Cooperative Oncology Group (ECOG) performance status of 0–2; (7) life expectancy greater than 3 months; (8) adequate organ function [white blood cell (WBC) count $\geq$ 4000 $\mu l^{-1}$, $\leq$ 10 000 $\mu l^{-1}$, absolute neutrophil count $\geq$ 2000 $\mu l^{-1}$, platelet count $\geq$ 100 000 $\mu l^{-1}$, haemoglobin $\geq$ 9.5 g $dl^{-1}$, transaminases level without liver metastases $\leq$ 2.5 times upper normal limit or with liver metastases $\leq$ 3 times upper normal limit, total bilirubin $\leq$ 1.5 mg $dl^{-1}$, serum albumin $\geq$ 3.0 g $dl^{-1}$, serum creatinine and blood urea nitrogen (BUN) $\leq$ upper normal limit, left ventricular ejection fraction measured by echocardiography $\geq$ 50%]; (9) age greater than 20 years and less than 75 years; and (10) no prior treatment with taxanes.

Exclusion criteria included: (1) a known history of a hypersensitive reaction to any drugs; (2) pregnancy and breast feeding; (3) active concomitant malignancy; (4) unfavourable medical conditions, such as uncontrolled infection, diabetes mellitus or cardiac disease; (5) prior bone marrow transplantation; (6) a peripheral neuropathy; (7) symptomatic brain metastases; and (8) hypercalcaemia (serum calcium $\geq$ 12 mg $dl^{-1}$). All patients gave written informed consent before registration.

### Drug administration and dose escalation procedures

Docetaxel and 5-FU were provided by Chugai Pharmaceutical, Tokyo, Japan and Rhone-Poulenc Rorer. Docetaxel was supplied as a concentrated sterile solution of 40 mg $ml^{-1}$ in polysorbate 80. This solution was diluted in 6 ml of 13% ethanol and then in 250 ml of 5% glucose and administered as a 1-h intravenous infusion on day 1. No premedication was performed for prevention of allergic reaction and emesis. 5-FU was diluted in 250–1000 ml per day of 5% glucose and administered after docetaxel as a continuous 24-h intravenous infusion on day 1 and then alone on days 2 to 5. Both drugs were administered using an electric infusion pump.

The starting doses of docetaxel and 5-FU were 40 mg $m^{-2}$ on day 1 and 150 mg $m^{-2}$ on days 1 to 5. Docetaxel/5-FU dose levels were escalated as follows: 40/300, 50/300, 50/500, 60/500 and 60/750 mg $m^{-2}$. We considered that dose-limiting toxicity (DLT) had been achieved if patients experienced at least one of the following: (1) grade 4 leucopenia or neutropenia lasting more than 5 days; (2) grade 2 fever lasting more than 3 days or sepsis proven by blood culture with grade 4 leucopenia or neutropenia; (3) grade 4 thrombocytopenia and (4) greater than grade 3 non-haematological toxicity. Intrapatient dose escalation was not allowed. It was planned to enter three patients at each level and if DLT was observed in one or two patients at one level, another three patients were entered at this level. The maximum-tolerated dose (MTD) was defined as the dosage that caused DLT in more than one half of the patients during the first course. Toxicity was evaluated according to Japan Clinical Oncology Group (JCOG) common toxicity criteria (Tobinai et al, 1993). Patients continued to receive their assigned treatment at the same dose level every 3–4 weeks, provided that they did not develop progressive disease, refuse further treatment or experience unacceptable toxicity. Patients who experienced unacceptable toxicity in a prior course were treated at a dosage-lowered level in the following courses.

### Evaluation and response criteria

Before the first course, each patient was assessed by physical examination, a complete medical history, chest radiography, bone radiography, including skull, vertebra and pelvis, ultrasound of the abdomen, computerized tomography of the brain, bone scintigram, electrocardiogram, echocardiography and routine laboratory studies; these consisted of a complete blood cell count, including differential WBC count, electrolyte, BUN, creatinine, glucose, total protein, serum albumin, calcium, alkaline phosphatase (Al-p), lactate dehydrogenase (LDH), total bilirubin, alanine aminotransferase (ALT), aspartate aminotransferase (AST) levels and urinalysis. The complete blood cell counts were repeated at

**Table 1** Patient characteristics

| | |
|---|---|
| Total no. of patients | 19 |
| No. of assessable patients | 18 |
| Age (years) | |
| Median (range) | 55 (34–72) |
| Performance status | |
| 0 | 8 |
| 1 | 8 |
| 2 | 2 |
| Advanced disease | 6 |
| Recurrent disease | 12 |
| No. of metastatic sites | |
| 1 | 3 |
| 2 | 6 |
| ≥3 | 9 |
| Site of disease | |
| Breast | 2 |
| Skin | 1 |
| Lymph node | 10 |
| Bone | 12 |
| Visceral | |
| Lung | 6 |
| Liver | 7 |
| Other viscera | 1 |
| Pleura/peritoneum | 4 |
| Prior chemotherapy | |
| Anthracycline (+) | 15 |
| Anthracycline (–) | 3 |
| Adjuvant only | 4 |
| Metastatic disease only | 6 |
| Both adjuvant and metastatic | 8 |
| Radiation therapy (local) | 6 |

© Cancer Research Campaign 1998

least three times per week after the start of treatment, and other examinations were repeated twice a week. Evaluation of response was required at each course. Response was evaluated according to Japanese Breast Cancer Society criteria (Japanese Breast Cancer Society, 1992). Complete response (CR) was defined as the disappearance of all clinical evidence of active tumour with complete reossification of bone lesions and absence of disease-related symptons for more than 4 weeks. Partial response (PR) was defined as more than 50% reduction in the sum of the products of two perpendicular diameters of all measurable lesions and remarkable reossification of osteolytic lesions without the appearance of new lesions, or less than 25% increase of any lesion for more than 4 weeks. No change (NC) was defined as a < 50% reduction or < 25% increase in the sum of the products of two perpendicular diameters of all measurable lesions and no remarkable improvement without the appearance of new lesions for at least 4 weeks and no increase of bone lesions without the appearance of new bone lesions for at least 8 weeks. Progressive disease (PD) was defined as the unequivocal appearance of any new lesions or more than 25% increase in the sum of perpendicular diameters of any measured lesion.

### Pharmacokinetic analysis

Heparinized blood samples (3 ml) for pharmacokinetic study were obtained from the arm not used for drug infusion. Samples were withdrawn at the following time points: before docetaxel infusion; 30 and 60 min after the start of docetaxel infusion; 15 and 30 min, 1, 2, 3, 4, 6 and 24 h after completion of docetaxel infusion on day 1, as well as before 5-FU infusion; 15 and 30 min, 1, 2, 3, 4, 6, 24, 48, 72, 96 and 120 h after the start of 5-FU infusion; and 15 and 30 min, 1, 2, 8 and 24 h after the completion of 5-FU infusion. Each blood sample was centrifuged immediately and the plasma

**Table 2** Results of treatment

| Level | Taxotere[a]/5-FU[b] | No. of patients/ no. of courses | Intolerable[c] |
|---|---|---|---|
| I | 40/150 | 3/20 | 0 |
| II | 40/300 | 3/23 | 0 |
| III | 50/300 | 3/18 | 0 |
| IV | 50/500 | 3/19 | 0 |
| V | 60/500 | 6/27 | 3 |

[a]mg m$^{-2}$ on day 1. [b]mg m$^{-2}$ on days 1 through 5. [c]No. of cases who showed dose-limiting toxicity.

was stored at –20°C until analysis. Docetaxel and 5-FU were assayed by high-performance liquid chromatography with UV detection (Jager et al, 1990; Vergniol et al, 1992). The detection limits for docetaxel and 5-FU were 7.5 and 10 ng ml$^{-1}$ respectively. The pharmacokinetic parameters of docetaxel were determined on the basis of non-compartment analysis. Total clearance (CL) was calculated by dividing the dose by the area under the concentration vs time curve (AUC). Volume of distribution at steady-state ($V_{ss}$) was estimated by the equation [CL × (mean residence time (MRT) – (Infusion time (h)/2)]. The half-life time ($t_{1/2}$) of docetaxel was determined using the residual method. The AUC of docetaxel and 5-FU was calculated using the trapezoidal method. The AUC, MRT and $t_{1/2}$ were calculated using the computer program MULTI (Yamaoka et al, 1981). In order to assess the pharmakokinetic and pharmakodynamic relationships of docetaxel and 5-FU, the percentage decrease in neutrophil count was calculated according to the formula: percentage decrease = [(pretreatment count – nadir count)/pretreatment count] × 100, and the relationships between this parameter and the AUC of docetaxel or 5-FU were analysed according to a sigmoid maximum effect ($E_{max}$) model (Holford and

**Table 3** Toxicity during the first course

| | Level I Grade[a] | | | | Level II Grade | | | | Level III Grade | | | | Level IV Grade | | | | Level V Grade | | | |
|---|---|---|---|---|---|---|---|---|---|---|---|---|---|---|---|---|---|---|---|---|
| | 1 | 2 | 3 | 4 | 1 | 2 | 3 | 4 | 1 | 2 | 3 | 4 | 1 | 2 | 3 | 4 | 1 | 2 | 3 | 4 |
| Leucopenia | 1 | 1 | 1 | | 2 | 1 | | | | 1 | 2 | | | 1 | 1 | | 1 | 2 | 1 | 2 |
| Neutropenia | | 1 | 1 | | 2 | 1 | | | | 1 | 1 | 1 | | | 1 | 1 | | 3 | | 3 |
| Thrombocytopenia | | | | | | | | | 1 | | | | | | | | | | | |
| Anaemia | 1 | 2 | | | | 2 | | | 3 | | | | | 1 | | | 1 | 4 | 1 | |
| Nausea and vomiting | 2 | | | | 1 | | | | 2 | | | | 1 | 1 | | | 2 | 3 | | |
| Diarrhoea | | 1 | | | 1 | 1 | | | 2 | | | | 1 | 1 | | | | 1 | 1 | 1 |
| Loss of appetite | 1 | | | | | 1 | | | 1 | | | | 1 | 1 | | | 3 | 1 | 1 | |
| Fatigue | | | | | 2 | 1 | | | | 1 | | | | 4 | 1 | | | | | |
| Stomatitis | 1 | | | | | 1 | | | | | | | 1 | | | | 2 | | | |
| Fever | 1 | 1 | | | | 2 | | | | | | | 1 | | | | 2 | 3 | | |
| Skin rash | 1 | | | | | | | | | | | | | | | | 1 | | | |
| Total bilirubin | | | | | | | | | | | | | | | | | | | | |
| LDH[b] | 1 | | | | | | | | 2 | | | | | | | | 3 | | | |
| Al-P[c] | 1 | | | | | | | | | | | | | | | | 1 | | | |
| Transaminases | 2 | | | | 1 | | | | | | | | 1 | | | | 5 | 1 | | |
| Hypoalbuminaemia | 1 | | | | 1 | | | | 2 | | | | 1 | | | | 2 | | | |
| Electrolyte imbalance | | | | | 1 | | | | | | | | 1 | | | | 1 | | 1 | |
| Vasculitis | | 1 | | | | | | | | | | | | 1 | | | | | | |
| Alopecia | 2 | | | | 3 | | | | 3 | | | | 3 | | | | 4 | 1 | | |
| Oedema (peripheral) | 1 | | | | 1 | | | | | | | | | | | | | | | |

[a]JCOG toxicity criteria. [b]Lactate dehydrogenase. [c]Alkaline phosphatase.

© Cancer Research Campaign 1998

Sheiner, 1982) as follows: percentage decrease in neutrophil count = $E_{max} \times (AUC)^r/[(AUC_{50})^r + (AUC)^r]$.

The maximum effect ($E_{max}$), $AUC_{50}$ (the AUC that produces 50% of the maximum effect) and $r$ (sigmoidicity coefficient) were estimated using non-linear least-squares regression using the computer program Win Nonlin (Scientific Consulting, Apex, NC, USA).

To determine the correlation between dose and the AUC or peak plasma concentrations ($C_{max}$) of docetaxel and 5-FU, Pearson correlation coefficients were calculated.

### RESULTS

### Patient characteristics

Nineteen patients were entered into this study from April to November, 1995. One patient was ineligible, because she had received two chemotherapy regimens after recurrence. Eighteen patients were evaluable for toxicity and response.

Patient characteristics are listed in Table 1. The number of patients and courses per dose level are listed in Table 2. The number of treatment courses of docetaxel/5-FU ranged from one to 13 (median, six; total, 107).

### Toxicity during the first course

#### *Haematological toxicity*

One of the major toxicities observed with this regimen was neutropenia (Table 3). One of three patients experienced grade 3 neutropenia at levels I, III and IV, while one of three patients experienced grade 4 neutropenia at levels III and IV. At level V, three of six patients experienced grade 4 neutropenia. Among these, one patient had grade 4 leucopenia, and neutropenia lasted for 5 days with grade 2 diarrhoea and fever; she subsequently received granulocyte colony-stimulating factor (G-CSF) support. Among most patients, neutrophil count reached its nadir on day 6 or 7 and recovered to 2000 $\mu l^{-1}$ by day 14. Mild anaemia was observed at each level and seemed to be dose related. One of six patients at level V experienced grade 3 anaemia.

#### *Non-haematological toxicity*

Gastrointestinal symptoms were the dominant non-haematological toxicity, with severity appearing to be dose related (Table 3). Diarrhoea was observed in one patient even at the lowest dose level, more than grade 1 diarrhoea was observed in each patient at dose levels I–IV. One of six patients at level V experienced grade 3 diarrhoea, which was observed on day 5 and was complicated with grade 2 fever. She recovered from diarrhoea on day 10. Another patient at the same level experienced grade 4 diarrhoea, which was observed on day 6 and was accompanied by grade 2 fever on day 10. Diarrhoea was improved by day 16. There was no relationship between the severity of diarrhoea and neutropenia. No severe hypersensitive reactions were observed. However, transient fever was observed on day 1 through 5 in two of 12 patients at levels I–IV, which was considered to be due to an allergic reaction. Only two patients at levels I and II had mild and transient oedema during the first course. Elevations of transaminases were observed in 4 of 12 patients, which appeared to be dose related. Five of ten patients with elevation of transaminases had liver metastases. Onset of these abnormalities ranged from between day 1 and day 15 (median day 5). Among ten patients with hepatic toxicity, six showed recovery within 14 days.

### Toxicity observed after more than two courses

Cumulative toxicities were not severe in all 18 patients who received more than two courses (Table 4). Anaemia seemed to be related to the number of treatment courses. Neutropenia, gastrointestinal toxicities and elevation of transaminases were not exacerbated by a greater number of treatment courses. Grade 1 or 2 elevation of transaminases was observed in 21 of 107 courses. One patient with three courses at level I and another with six courses at level III had grade 1 peripheral oedema. At level V, one patient with two courses and another patient with three courses had grade 1 and transient peripheral oedema. One patient treated with more than two courses at level I and another with more than nine courses at level II had mild and transient paraesthesia. Four of 18 patients who received more than two courses experienced grade 1 or 2 vasculitis. Dose reductions of one level were mandated in the three of six patients at level V because of grade 3 diarrhoea or grade 4 neutropenia during the first course.

### Therapeutic efficacy

All patients had measurable disease. One patient with lymph node metastases achieved CR and eight patients achieved PR (two patients with lymph node metastases, two with lymph node and

**Table 4** Toxicity of all courses

| | Level I Grade[a] | | Level II Grade | | Level III Grade | | Level IV Grade | | Level V Grade | |
|---|---|---|---|---|---|---|---|---|---|---|
| | 1 or 2 | ≥3 | 1 or 2 | ≥3 | 1 or 2 | ≥3 | 1 or 2 | ≥3 | 1 or 2 | ≥3 |
| Neutropenia | 4 | 11 | 12 | 3 | 7 | 11 | 9 | 5 | 6 | 15 |
| Thrombocytopenia | | | 2 | | 2 | | | | | |
| Anaemia | 17 | | 10 | | 8 | | 6 | | 26 | 1 |
| Diarrhoea | 4 | | 7 | | 7 | | 3 | | 4 | 3 |
| Stomatitis | 4 | | 3 | | | | 1 | | 4 | |
| Fever | 3 | | 2 | | | | | 2 | 11 | |
| Transaminases | 3 | | 3 | | 3 | | 5 | | 11 | |
| Paresthesia | 2 | | 3 | | | | | | | |
| Vasculitis | 2 | | | | 1 | | 1 | | 1 | |
| Oedema (peripheral) | 2 | | 1 | | 1 | | | | 3 | |

[a]JCOG toxicity criteria.

© Cancer Research Campaign 1998

**Table 5** Therapeutic efficacy

| Level | Taxotere[a]/5-FU[b] | Response |
|---|---|---|
| I | 40/150×5 days | 1 PR, 1 NC, 1 PD |
| II | 40/300×5 days | 2 PR, 1 PD |
| III | 50/300×5 days | 1 CR, 1 NC, 1 PD |
| IV | 50/500×5 days | 2 PR, 1 NC |
| V | 60/500×5 days | 3 PR, 1 NC, 2 PD |

[a]mg m$^{-2}$ on day 1. [b]mg m$^{-2}$ on days 1 to 5. CR, complete response; PR, partial response; NC, no change; PD, progressive disease.

bone, two with liver, one with lung and liver, and one with bone). The response rate was 50% (95% CI 26–74%) (Table 5). Seven of nine patients who showed response in the present combination had been treated with anthracycline-containing regimens previously.

### Pharmacokinetics and pharmacodynamics

Plasma concentrations of docetaxel and 5-FU were quantitated in 18 and 17 patients, respectively, during the first course. Pharmacokinetic parameters of docetaxel and 5-FU are summarized in Table 6A and B. Mean plasma concentration–time profiles are illustrated in Figure 1A and B. Plasma disappearances of docetaxel showed biphasic or triphasic profiles. The interpatient variations in plasma concentrations of docetaxel and 5-FU were large. $C_{max}$ and AUC of docetaxel were poorly related to the dose of docetaxel ($r$ = 0.08, $P$ = 0.96, and 0.01, $P$ = 0.77, respectively). $C_{max}$ and AUC of 5-FU increased in proportion to the dose of 5-FU ($r$ = 0.75, $P$ < 0.01, and 0.81, $P$ < 0.01, respectively). The relationship between the AUC of docetaxel and the percentage decrease in neutrophil count during the first course was approximated using a sigmoid $E_{max}$ model ($E_{max}$ = 87.2±7.2%, $AUC_{50}$ = 874.8±95.3 ng h ml$^{-1}$ and $r$ = 3.3±1.5). But there was no significant relation between the AUC of 5-FU and the percentage decrease in neutrophil count. Scatterplots depicting the grade of diarrhoea vs the AUC and $C_{max}$ of docetaxel and 5-FU during the first course did not indicate a significant relation.

### DISCUSSION

It is of cardinal importance to conduct clinical trials to evaluate combination regimens including docetaxel to determine whether they have enhanced anti-tumour efficacy. In the present study, we chose continuous 5-FU infusion in combination with docetaxel to evaluate clinical synergistic anti-tumour effects as a second-line chemotherapy for advanced or recurrent breast cancer. We decided to test docetaxel and 5-FU in doses of 40–60 mg m$^{-2}$ and 150–500 mg m$^{-2}$ day$^{-1}$ respectively.

In the Japanese phase II trials of docetaxel administered at 60 mg m$^{-2}$ as a 1-h infusion every 3 or 4 weeks for previously treated MBC, 61 of 71 patients (85.9%) and 56 of 66 (84.8%) showed grade 3 and 4 neutropenia (Taguchi et al, 1994*b*; Adachi et

**Table 6** Mean (± s.d.) pharmacokinetic parameters of docetaxel (A) and 5-FU (B) at the first course

A

| Dose level Docetaxel/5-FU×5 days (mg m$^{-2}$) | No. of patients | $C_{max}$ (ng ml$^{-1}$) | $t_{1/2}$ (h)[a] | AUC (0–∞) (ng h ml$^{-1}$) | CL (mg m$^{-2}$) | $V_{ss}$ (mg m$^{-2}$) |
|---|---|---|---|---|---|---|
| 40/150 | 3 | 1354.4 ± 482.7 | γ = 10.9, 15.8<br>β = 0.7 | 1801.3 ± 810.3 | 25.0 ± 9.5 | 95.2 ± 79.3 |
| 40/300 | 3 | 1838.8 ± 830.8 | γ = 12.3<br>β = 4.0, 1.0 | 2316.0 ± 1859.0 | 24.6 ± 13.7 | 33.8 ± 11.3 |
| 50/300 | 3 | 1222.4 ± 353.9 | γ = 25.1<br>β = 1.3, 3.6 | 1293.5 ± 398.6 | 41.8 ± 15.7 | 175.7 ± 241.5 |
| 50/500 | 3 | 1462.3 ± 369.0 | γ = 5.6 ± 1.6[b] | 1599.1 ± 441.8 | 33.2 ± 10.4 | 51.2 ± 30.2 |
| 60/500 | 6 | 1678.0 ± 376.4 | γ =20.0 ± 21.5[b]<br>β =2.3 ± 1.2*[b] | 2241.6 ± 955.0 | 30.7 ± 11.6 | 116.6 ± 170.6 |

B

| Dose level Docetaxel/5-FU×5days (mg m$^{-2}$) | No. of patients | $C_{max}$ (ng ml$^{-1}$) | AUC (0–*t*) (ng h ml$^{-1}$) |
|---|---|---|---|
| 40/150 | 3 | 66.1 ± 14.0 | 4613.0 ± 879.1 |
| 40/300 | 3 | 143.9 ± 19.8 | 9940.2 ± 3323.3 |
| 50/300 | 3 | 161.3 ± 72.3 | 13603.5 ± 5737.2 |
| 50/500 | 3 | 385.0 ± 66.0 | 24633.4 ± 7282.8 |
| 60/500 | 5 | 376.3 ± 218.2 | 26103.5 ± 9986.4 |

[a]Terminal half-life time. [b]$n$=3. $C_{max}$, peak plasma concentration; AUC, area under concentration vs time curve; CL, total clearance; $V_{ss}$, volume of distribution at steady-state.

© Cancer Research Campaign 1998

**Figure 1** (**A**) Mean plasma concentration curve of docetaxel. (**B**) Mean plasma concentration curve of 5-FU

al, 1996). In the phase I trial of a single infusion of docetaxel as a 1-h infusion, two of six patients experienced grade 3 and 4 neutropenia at a dose of 50 mg $m^{-2}$ and three of six experienced grade 3 at a dose of 60 mg $m^{-2}$ (Ringel and Horwitz, 1991). Taking the neutropenic profile of these trials of a single infusion of docetaxel, combination of a 5-day continuous infusion of 5-FU with docetaxel dose not seem to exacerbate neutropenia.

Severe diarrhoea was not frequently observed in a single 1-h infusion of docetaxel phase II trials. In the Japanese phase II trials, one of 71 patients and one of 66 patients showed grade 3 diarrhoea (Taguchi et al, 1994*b*; Adachi et al, 1996). In the phase II trials of docetaxel administered at 100 mg $m^{-2}$, the frequencies of greater than grade 2 diarrhoea were 7–15% (Chevallier et al, 1995; Valero et al, 1995; Hudis et al 1996). On the other hand, it has been reported that 5-FU frequently induces mild to moderate diarrhoea. Frequency and severity of 5-FU-induced diarrhoea became much less severe when given as a continuous infusion. In a study of 5-FU administered by a 5-day continuous infusion schedule for patients with metastatic colorectal cancer, when 26 patients received this treatment at doses ranging from 650 to 1300 mg $m^{-2}$ day,$^{-1}$ grade 1 diarrhoea was observed in only one of a total of 185 courses (Milano et al, 1988). The diarrhoea observed in the present combination, therefore, seems to be extraordinarily frequent and severe. This excarbation may be the result of a possible drug–drug interaction of docetaxel and 5-FU. Further investigation is needed to elucidate the mechanism of this adverse synergy.

In the previous phase I and II trials of a 1-h infusion of docetaxel, mild hepatic toxicity was observed in approximately 50% of the patients (Hudis et al, 1996); nor was this toxicity remarkable in patients with 5-FU (Milano et al, 1988). The higher incidence of hepatic toxicity observed in the present trial denotes the need to clarify the interactions between these two agents at the metabolic pathways.

In the previous studies of docetaxel administered as a single infusion, it was reported that the AUC and the percentage decrease in neutrophil count fitted well with a sigmoid $E_{max}$ model (Bissett et al, 1993; Extra et al, 1993). In the pharmacokinetic analysis of the present combination of docetaxel and 5-FU, the AUC of docetaxel correlated with the percentage decrease in neutrophil count in this model. But .a significant relationship between the AUC of 5-FU and the percentage decrease in neutrophil count was not observed. These findings may indicate that docetaxel is the principal contributor to neutropenia in the present combination. And no remarkable relationship between pharmacokinetic parameters of docetaxel or 5-FU and grade of diarrhoea were observed.

In this study, three of six patients showed DLTs (one with grade 4 neutropenia that lasted for 5 days and two with grade 3 and 4 diarrhoea). Therefore, we considered that the MTD was 60 mg $m^{-2}$ of docetaxel on day 1 and 500 mg $m^{-2}$ per day of 5-day continuous infusion of 5-FU.

Seven of 15 patients who were previously treated with anthracycline-containing chemotherapy achieved responses. This regimen therefore seems to be active as a second-line chemotherapeutic regimen against MBC. The activity of this regimen against MBC should be evaluated further in a phase II trial.

In conclusion, 50 mg $m^{-2}$ of docetaxel over 1 h on day 1 and 500 mg $m^{-2}$ $day^{-1}$ of continuous infusion of 5-FU on days 1 to 5 at 3- or 4-week intervals is an appropriate schedule for future phase II trials in patients previously treated for MBC. This combination seems to be active as a second-line chemotherapeutic regimen against MBC. Care should be taken to manage diarrhoea, which may be adversely enhanced by this combination.

### ACKNOWLEDGEMENTS

We thank Dr Takashi Fukutomi, Dr Tomio Wada and Dr Yuichi Takatsuka for evaluation of therapeutic response in extramural reviews; Mr Sizuhiro Yamada, Miss Rieko Uemura and Chugai Pharmaceutical for their reliable data management; Mr Masaki Kashimura and Rhone-Poulenc Rorer Japan Ibaraki Laboratory for assays of docetaxel and 5-FU; and Mr Takeshi Ueno and Dr Nacer Azli, Rhoune-Poulenc Rorer for reviewing this paper. This study was supported by Chugai Pharmaceutical, Tokyo, Japan.

### REFERENCES

Adachi I, Watanabe T, Takashima S, Narabayashi M, Horikoshi N, Aoyama H and Taguchi T (1996) A late phase II study of RP56976 (docetaxel) in patients with advanced or recurrent breast cancer. *Br J Cancer* **73**: 210–216

Ansfield FJ, Ramizes G, Mackman S, Bryan GT and Curreri AR (1969) A 10-year study of 5-fluorouracil in disseminated breast cancer with clinical results and survival times. *Cancer Res* **29**: 1062–1066

Bissery MC, Vrignard P, Bayssas M and Lavelle F (1993) Taxotere synergistic combination with cyclophosphamide, etoposide and 5-fluorouracil in mouse tumor models (abstract). *Proc Am Assoc Cancer Res* **34**: 299

© Cancer Research Campaign 1998

Bissett D, Setanoians A, Cassidy J, Graham MA, Chadwick GA, Wilson P, Auzannet V, Bail NL and Kane SB (1993) Phase I and pharmacokinetic study of Taxotere (RP 56976) administered as a 24-hour infusion. *Cancer Res* **53**: 523–527

Cameron DA, Gabra H and Leonard RCF (1994) Continuous 5-fluorouracil in the treatment of breast cancer. *Br J Cancer* **70**: 120–124

Chevallier B, Fumoleau P, Kerbrat P, Dieras V, Roche H, Krakowski I, Azli N, Bayssas M, Lentz MA and Van Glabbeke M (1995) Docetaxel is a major cytotoxic drug for the treatment of advanced breast cancer: a phase II trial of the Clinical Screening Cooperative Group of the European Organization for Research and Treatment of Cancer. *J Clin Oncol* **13**: 314–322

DeVita VT, Hellman S and Rosenberg SA (1993) *Cancer (Principles & Practice of Oncology)*, 4th edn. Lippincott: Philadelphia. pp. 1315–1322

Extra JM, Rousseau F, Bruno R, Clavel M, Bail NL and Marty M (1993) Phase I and pharmacokinetic study of Taxotere (RP 56976; NSC 628503) given as a short intravenous infusion. *Cancer Res* **53**: 1037–1042

Gregory WM, Smith P, Richards MA, Twelves CJ, Knight RK and Rubens RD (1993) Chemotherapy of advanced breast cancer: outcome and prognostic factors. *Br J Cancer* **68**: 988–995

Holford NH and Sheiner LB (1982) Kinetics of pharmacologic response. *Pharmacol Ther* **16**: 143–166

Hudis CA, Seidman AD, Crown JPA, Balmaceda C, Freilich R, Gilewski TA, Hakes TB, Currie V, Lebwohl DE, Baselga J, Raptis G, Gollub M, Robles M, Bruno R and Norton L (1996) Phase II and pharmacologic study of docetaxel as initial chemotherapy for metastatic breast cancer. *J Clin Oncol* **14**: 58–65

Jabboury K, Holmes FA and Hortobagyi GN (1989) 5-Fluorouracil rechallenge by protracted infusion in refractory breast cancer. *Cancer* **64**: 793–797

Jager W, Czejka MJ, Schuller J, Fogl U, Czejka E and Lackner H (1990) Rapid and simple high-performance liquid chromatographic assay for 5-fluorouracil in plasma for bioavailability studies. *J Chromatogr* **532**: 411–417

Japanese Breast Cancer Society (1992) *General Rules for Clinical and Pathological Recording of Breast Cancer* (in Japanese), 11th edn. pp. 53–61. Kanahara: Japan

Lavelle F, Bissery MC, Combeau C, Riou JF, Vrignard P and Andre S (1995) Preclinical evaluation of docetaxel (Taxotere). *Semin Oncol* **22** (suppl. 4): 3–16

Milano G, Roman P, Khater R, Frenay M, Renee N and Namer M (1988) Dose versus pharmacokinetics for predicting tolerance to 5-day continuous infusion of 5-FU. *Int J Cancer* **41**: 537–541

Ravdin PM, Burris III HA, Cook G, Eisenberg P, Kane M, Bierman WA, Mortimer J, Genevois E and Bellet RE (1995) Phase II trial of docetaxel in advanced anthracycline-resistant or anthracenedione-resistant breast cancer. *J Clin Oncol* **13**: 2879–2885

Ringel I and Horwitz SB (1991) Studies with RP56976: a semisynthetic analogue of Taxol. *J Natl Cancer Inst* **83**: 288–291

Taguchi T, Furue H, Niitani H, Ishitani K, Kanamaru R, Hasegawa K, Ariyoshi H, Noda K, Furuse K, Fukuoka M, Yakushiji M and Kashimura M (1994*a*) Phase I clinical trial of RP 56976 (docetaxel) a new anticancer drug (in Japanese). *Jpn J Cancer Chemother* **21**: 1997–2005

Taguchi T, Mori S, Abe R, Hasegawa K, Morishita Y, Tabei T, Sasaki Y, Fujita M, Enomoto K, Hamano K, Tominaga T, Sasaki T, Yamaguchi S, Nishiyama K, Iida F, Kanda K, Takagi H, Masaoka A, Takahashi T, Oka T, Takai S, Ota J, Wada T, Yayoi K, Naito Y, Konishi Y, Sonoo H, Hiraki S, Yamamoto Y, Nakase A, Dohi K, Monden Y and Ogawa M (1994*b*) Late phase II clinical study of RP56976 (docetaxel) in patients with advanced/recurrent breast cancer (in Japanese). *Jpn J Cancer Chemother* **21**: 2625–2632

Tobinai K, Kohno A, Shimada Y, Watanabe T, Tamura T, Takeyama K, Narabayashi M, Fukutomi T, Kondo H, Shimoyama M and Suemasu K (1993) Toxicity grading criteria of the Japanese Clinical Oncology Group. *Jpn J Clin Oncol* **23**: 250–257

Trudeau ME, Eisenhauer E, Lofters W, Norris B, Muldal A, Letendre F, Vandenburg T and Verma S (1993) Phase II study of Taxotere as first line chemotherapy for metastatic breast cancer (MBC). A National Cancer Institute of Canada Clinical Trials Group (NCIC CTG) study. *Proc Am Soc Clin Oncol* **12** (abstract 59)

Valero V, Holmes FA, Walters RS, Theriault RL, Esparza L, Fraschini G, Fonseca GA, Bellet RE, Buzdar AU and Hortobagyi GN (1995) Phase II trial of docetaxel: a new, highly effective antineoplastic agent in the management of patients with anthracycline-resistant metastatic breast cancer. *J Clin Oncol* **13**: 2886–2894

Vergniol JC, Bruno R, Montay G and Frydman A (1992) Determination of Taxotere in human plasma by a semi-automated high-performance liquid chromatographic method. *J Chromatogr* **582**: 273–278

Yamaoka K, Tanigawara Y and Nakagawa T (1981) A pharmacokinetic analysis program (multi) for microcomputer. *J Pharmacobio-Dyn* **4**: 879–885

© Cancer Research Campaign 1998